# Prion Protein Protects against Renal Ischemia/Reperfusion Injury

**DOI:** 10.1371/journal.pone.0136923

**Published:** 2015-09-01

**Authors:** Bo Zhang, Daniel Cowden, Fan Zhang, Jue Yuan, Sandra Siedlak, Mai Abouelsaad, Liang Zeng, Xuefeng Zhou, John O'Toole, Alvin S. Das, Diane Kofskey, Miriam Warren, Zehua Bian, Yuqi Cui, Tao Tan, Adam Kresak, Robert E. Wyza, Robert B. Petersen, Gong-Xian Wang, Qingzhong Kong, Xinglong Wang, John Sedor, Xiongwei Zhu, Hua Zhu, Wen-Quan Zou

**Affiliations:** 1 Institute of Organ Transplantation, Tongji Hospital, Tongji Medical College, Huazhong University of Science and Technology, Wuhan, HuBei, The People’s Republic of China; 2 Department of Pathology, Case Western Reserve University/University Hospitals Case Medical Center, Cleveland, Ohio, United States of America; 3 Department of Surgery, Davis Heart and Lung Research Institute, The Ohio State University, Columbus, Ohio, United States of America; 4 Department of Urology, The First Affiliated Hospital, Nanchang University, Nanchang, Jiangxi Province, The People’s Republic of China; 5 National Prion Disease Pathology Surveillance Center, Case Western Reserve University/University Hospitals Case Medical Center, Cleveland, Ohio, United States of America; 6 Kidney Disease Research Center, Case Western Reserve University, Cleveland, Ohio, United States of America; 7 Department of Neurology, Case Western Reserve University/University Hospitals Case Medical Center, Cleveland, Ohio, United States of America; 8 National Center for Regenerative Medicine, Case Western Reserve University/University Hospitals Case Medical Center, Cleveland, Ohio, United States of America; 9 Departments of Medicine and Physiology and Biophysics, Case Western Reserve University, Cleveland, Ohio, United States of America; 10 Human Tissue Procurement Facility (HTPF) and the Comprehensive Cancer Center Tissue Resources Core, Case Western Reserve University/University Hospitals Case Medical Center, Cleveland, Ohio 44106, United States of America; 11 Key Laboratory of Ministry of Health and Key Laboratory of Ministry of Education, Wuhan, HuBei, The People’s Republic of China; 12 Department of Neuroscience, Case Western Reserve University/University Hospitals Case Medical Center, Cleveland, Ohio, United States of America; 13 Department of Neurosurgery, Shandong University, Jinan, The People’s Republic of China; 14 State Key Laboratory for Infectious Disease Prevention and Control, National Institute for Viral Disease Control and Prevention, Chinese Center for Disease Control and Prevention, Beijing, The People’s Republic of China; Rocky Mountain Laboratories, NIAID, NIH, UNITED STATES

## Abstract

The cellular prion protein (PrP^C^), a protein most noted for its link to prion diseases, has been found to play a protective role in ischemic brain injury. To investigate the role of PrP^C^ in the kidney, an organ highly prone to ischemia/reperfusion (IR) injury, we examined wild-type (WT) and PrP^C^ knockout (KO) mice that were subjected to 30-min of renal ischemia followed by 1, 2, or 3 days of reperfusion. Renal dysfunction and structural damage was more severe in KO than in WT mice. While PrP was undetectable in KO kidneys, Western blotting revealed an increase in PrP in IR-injured WT kidneys compared to sham-treated kidneys. Compared to WT, KO kidneys exhibited increases in oxidative stress markers heme oxygenase-1, nitrotyrosine, and N^ε^-(carboxymethyl)lysine, and decreases in mitochondrial complexes I and III. Notably, phosphorylated extracellular signal-regulated kinase (pERK) staining was predominantly observed in tubular cells from KO mice following 2 days of reperfusion, a time at which significant differences in renal dysfunction, histological changes, oxidative stress, and mitochondrial complexes between WT and KO mice were observed. Our study provides the first evidence that PrP^C^ may play a protective role in renal IR injury, likely through its effects on mitochondria and ERK signaling pathways.

## Introduction

Ischemia-reperfusion (IR) is the leading cause of acute kidney injury (AKI) and is often associated with significant morbidity and mortality in a variety of clinical situations such as renal transplantation, renal artery reconstruction, cardiac arrest, and shock [1–4]. Renal IR injury is mainly characterized by acute tubular damage manifested by loss of the brush border, tubular dilatation/vacuolation, tubular apoptosis and necrosis [5,6]. Several mechanisms have been proposed for renal IR injury. For instance, oxidative stress is believed to play an important role in IR-induced kidney injury [2,7,8], in addition to reduced glomerular filtration and accumulation of leukocytes [9]. Specifically, both generation of reactive oxygen/nitrogen species and the loss of antioxidant defense mechanisms have been implicated in the pathogenesis of IR-induced tissue injury. Given our incomplete understanding of the mechanisms underlying IR-associated AKI, elucidation of additional factors involved in modulating the oxidant/antioxidant balance in animals and humans may provide insights into the pathogenesis of AKI.

The cellular prion protein (PrP^C^) is a cell-surface copper-binding glycoprotein not only expressed in the central nervous system, but also in peripheral organs including the kidney [10–14]. A misfolded, infectious isoform of prion protein (PrP^Sc^) that arises from a conformational transition of PrP^C^ has gained notoriety due to its association with prion diseases [15,16]; however, the physiological function of PrP^C^ remains poorly understood. Several lines of evidence have shown that PrP^C^ plays multiple roles in neuronal and non-neuronal cells, including cellular trafficking [17,18], copper uptake [19], cell adhesion/differentiation [20], cell signaling [21], and neuronal survival [22]. PrP^C^ has also been observed to protect neural tissues against oxidative stress [23,24]. PrP^C^ expression is upregulated after focal cerebral ischemia *in vivo*, which was associated with lesion severity [25]. Moreover, PrP^C^ expression is increased in neurons of ischemic human and animal brains and the infarct size in PrP-knockout (KO) mice is significantly larger than that observed in wild-type (WT) mice [26]. In addition, Shyu et al showed that overexpression of PrP^C^ by adenovirus-mediated gene transfer could reduce ischemic injury and improve neurological dysfunction after cerebral ischemia in rats [27]. These findings along with others [28–31] clearly indicate that PrP^C^ plays a protective role during ischemic brain injury.

Does the protein play a protective role in the peripheral organs such as the kidney and heart that are particularly susceptible to ischemic injury? A recent *in vitro* study reported that overexpression of PrP^C^ reduced cardiac oxidative stress and cell death caused by post-ischemic reperfusion; conversely, deletion of PrP^C^ increased oxidative stress during both ischemic preconditioning and perfusion with hydrogen peroxide [32]. To confirm and extend these findings, in the current study, we used an *in vivo* model to compare the changes in renal function and histology resulting from 30-minute of ischemia followed by one, two, or three days of reperfusion as well as the proteins associated with oxidative stress, mitochondrial respiratory chain complexes, and IR-related signaling pathways.

## Results

### More severe acute renal failure is observed in KO than in WT mice subjected to ischemia-reperfusion injury

**Fig 1:**
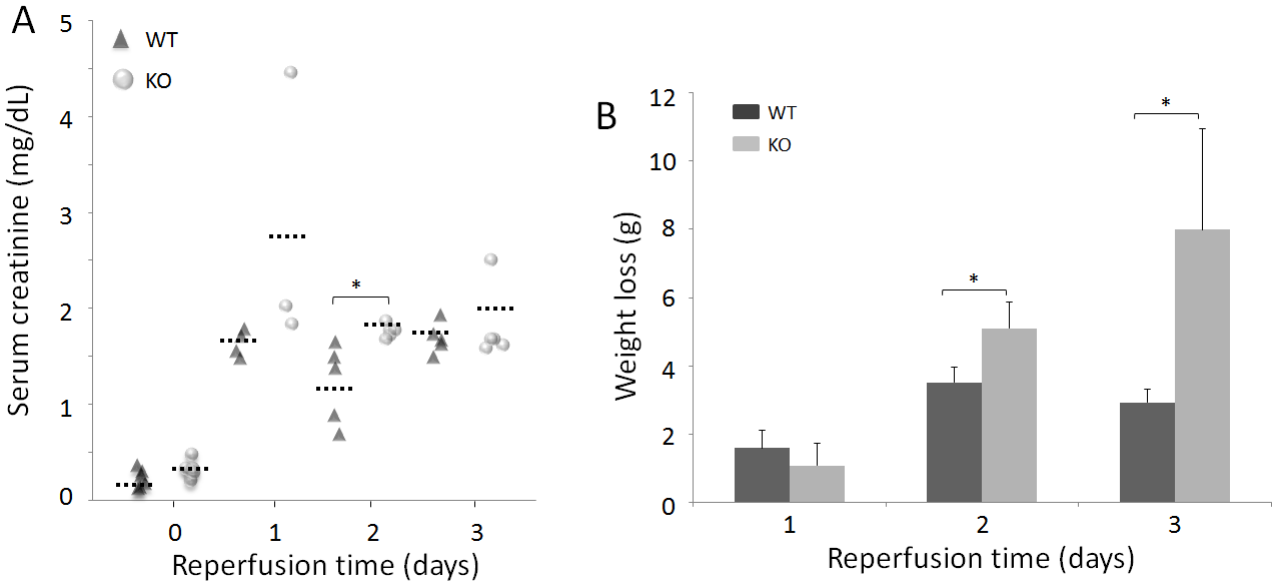
Serum creatinine and weight loss of sham and IR-injured WT and KO mice. (A) Comparison of serum creatinine of sham and IR-injured mice. The solid black triangles (▲) represent WT mice, whereas the solid gray circles (●) represent KO mice. The short dotted-lines (**…‥**) represent the mean creatinine values of each group of mice. WT: Day 0 (sham mice, n = 6); Day 1 (n = 4); Day 2 (n = 5); and Day 3 (n = 5). KO: Day 0 (sham, n = 6); Day 1 (n = 3); Day 2 (n = 5); and Day 3 (n = 5). (B) Comparison of body weight loss between mice subjected to 1, 2, or 3 days of reperfusion. WT: Day 1 (n = 4); Day 2 (n = 3); Day 3 (n = 3). KO: Day 1 (n = 3); Day 2 (n = 5); Day 3 (n = 4). *p < 0.05.

In the sham animals, the average serum creatinine concentration was smaller in WT than in KO mice but the difference was not statistically significant [0.23 ± 0.09 mg/dL (n = 6) vs. 0.33 ± 0.08 mg/dL (n = 6), *p* = 0.14 > 0.05]. After removal of the right side of the kidney, both KO and WT mice were subjected to left renal pedicle clamping for 30 min followed by reperfusion for 1, 2, or 3 days before sacrificed. As expected, the animals exhibited elevated serum creatinine and decreased body weight (Fig 1A and 1B). Compared to sham mice, the levels of serum creatinine were significantly increased at all three time points in IR-injured WT and KO mice and the differences in the means of serum creatinine were highly significant among all different groups of WT mice (*p* < 0.001), KO mice (*p* < 0.001), or all groups from both WT and KO (*p* < 0.001), as indicated by one-way analysis of variance (ANOVA). Notably, the levels of serum creatinine in KO mice were significantly higher than in WT mice on day 2 (Fig 1A and Table 1). Although a difference in the level of serum creatinine was observed between KO and WT mice on day 1, it was not statistically significant, which may be due to the possibility of the small number of animal used and high variation (Fig 1A). Also, there was no significant difference in the level of serum creatinine between KO and WT mice on day 3 (Table 1).

**Table 1:** Serum creatinine of ischemia/reperfusion-injured and sham mice.

Animal strains	Sham (mg/dL)	IR-Day 1 (mg/dL)	IR-Day 2 (mg/dL)	IR-Day 3 (mg/dL)
**Wild-type (n)**	0.23 ± 0.09 (6)	1.65 ± 0.14*** (4)	1.23 ± 0.41* Δ (5)	1.70 ± 0.16**** (5)
**Knockout (n)**	0.33 ± 0.08 (6)	2.77 ± 1.46 (3)	1.76 ± 0.07**** (5)	1.81 ± 0.39** (5)

*p <0.05

**p < 0.01

***p < 0.005

****p < 0.001 compared with sham mice

^Δ^ *p* < 0.05 compared with knockout mice on day 2; IR, Ischemia/reperfusion injury.

The one-way ANOVA exhibited that the differences in the means of body weight loss were highly significant among all groups of IR-injured WT and KO mice (*p* < 0.001), or among all three IR-injured WT (*p* < 0.005), or KO mice (*p* < 0.005). Moreover, loss of body weight was more significant on day 2 in KO than in WT mice (Fig 1B and Table 2). Compared to day 1, the weight loss increased with time and was significantly greater in KO mice compared to WT 2 and 3 days after IR-injury (Fig 1B and Table 2).

**Table 2:** Loss of body weight of IR-injured mice.

Mouse line	IR-Day 1 (g)	IR-Day 2 (g)	IR-Day 3 (g)
**Wild-type (n)**	1.6 ± 0.52 (4)	3.5 ± 0.44*** Δ (3)	2.9 ± 0.38* Δ (3)
**Knockout (n)**	1.4 ± 0.65 (3)	5.1 ± 0.75**** (5)	8.0 ± 2.97** (4)

*p <0.05

**p < 0.01

***p < 0.005

****p < 0.001 compared with mice on day 1

^Δ^ *p* < 0.05 compared with knockout mice on day 2, or 3; IR, Ischemia/reperfusion injury.

### More tubular damage is observed in KO than in WT kidneys subjected to IR injury

**Fig 2:**
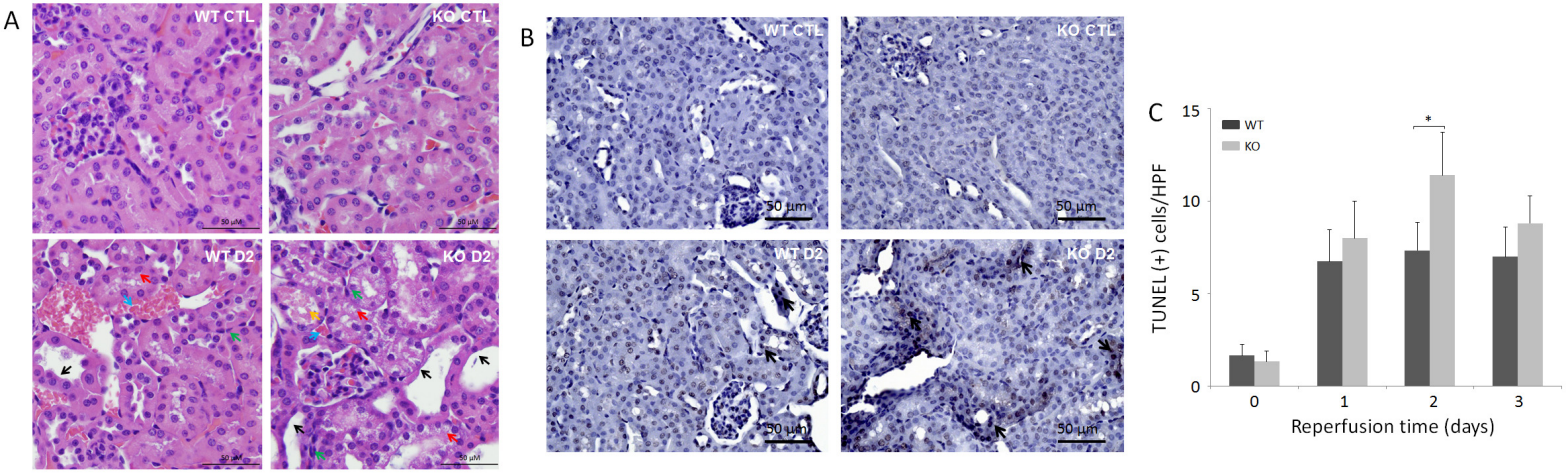
Histological changes and TUNEL apoptosis assays of sham and IR-injured WT and KO mice. (A) Hematoxylin and eosin (H&E) staining images are representative renal tissue sections from three mice of each group including WT and KO control (CTL) and IR-injured on day 2 (bar = 50 μm; original magnification, x 400). Black arrows indicate dilated tubules and brush-border damage. Red arrows indicate tubular vacuolization. Blue arrows indicate peritubular capillary congestion. Orange arrow indicates cast formation. Green arrows indicate necrotic changes, such as the frequent presence of pyknotic nuclei. (B) TUNEL apoptosis images are representative renal tissue sections from three mice of each group including WT and KO control (CTL) and IR-injured on day 2 (bar = 50 μm; original magnification, x 400). Black arrows indicate apoptotic cells with brown staining. (C) Quantitative analysis of apoptotic cells on day 0, 1, 2, and 3. Apoptotic cells were quantified by counting TUNEL-positive cells in 4 random high-power fields (HPFs, x 400). *p < 0.05.

To determine whether renal dysfunction induced by IR injury was associated with renal pathologic damage, hematoxylin & eosin (H&E) staining of the kidney was performed. Both WT and KO mice displayed tubular damage on days 1, 2, and 3 after renal IR injury, as evidenced by tubular dilatation/vacuolation, loss of the brush border, cast formation, tubular necrosis, and peritubular capillary congestion at the site of the renal cortex or corticomedullary junction (Fig 2A). Consistent with the renal dysfunction described above, more significant pathological changes were observed in KO than in WT kidneys, especially on day 2. The renal medulla in kidneys from sham KO and WT was largely unaffected (Fig 2A).

An *in situ* terminal deoxynucleotidyl transferase dUTP nick end labeling (TUNEL) assay was subsequently performed to determine whether apoptosis occurred (Fig 2B). Compared to sham WT or KO mice, the number of TUNEL-positive cells in IR-injured WT or KO mice was dramatically increased on day 1, 2 or 3, respectively. The one-way ANOVA indicated that the differences in the means of the number of apoptotic cells were highly significant among all groups of WT and KO mice (*p* < 0.001), among four groups of WT mice (*p* < 0.005, and among four groups of KO mice (*p* < 0.001). Notably, more apoptotic tubular cells were observed in KO than in WT mice, especially on day 2 (*p* < 0.05) (Fig 2C).

### The levels of PrP are significantly higher in IR-injured than in sham kidneys of WT mice

**Fig 3:**
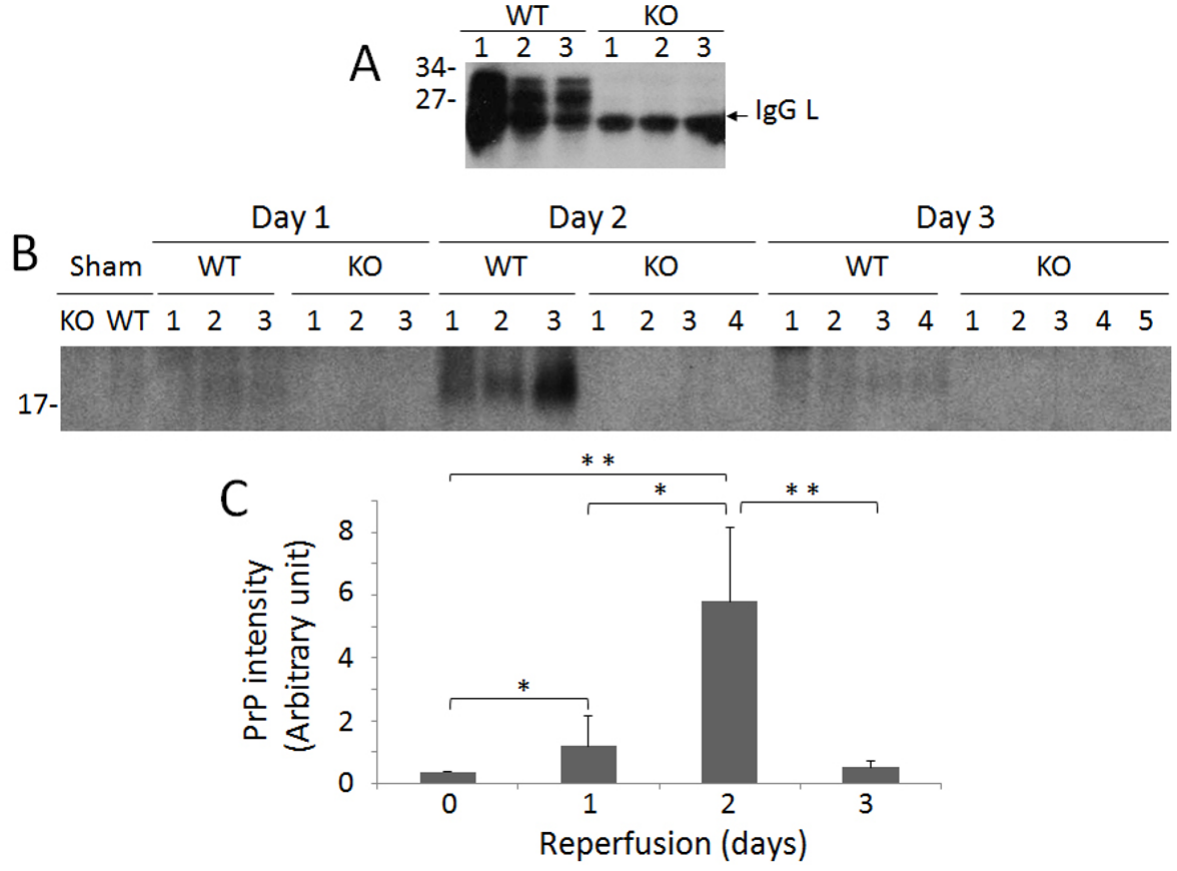
Detection of PrP^C^ in the frozen kidney tissues of sham and IR-injured WT and KO mice. (A) PrP^C^ was detected in the frozen renal tissues from three IR-injured WT and KO mice each with two days of reperfusion by Western blotting probing with 6D11. A band migrating at ~25 kDa detected in both KO and WT kidneys is IgG light chain. (B) PrP^C^ was detected in the frozen renal tissues from sham and IR-injured WT and KO mice with 1, 2 or 3 days of reperfusion by Western blotting probing with 6D11 after treated with PNGase F to remove N-linked glycans. The PrP band migrating at ~18 kDa corresponds to C1. (C) The Western blots are representative of three experiments. The intensity of PrP^C^ band from the kidney of sham and IR-injured mice was quantitated by densitometric analysis. WT: Day 1 (n = 3); Day 2 (n = 3); Day 3 (n = 4). KO: Day 1 (n = 3); Day 2 (n = 4); Day 3 (n = 5). *p < 0.05; **p < 0.01.

PrP^C^ in the kidney was detected by Western blotting using the anti-PrP antibody 6D11. While no PrP was detectable in the KO kidney, a significant amount of PrP was readily detected in the WT kidney subjected to ischemia followed by reperfusion for two days (Fig 3A). There was a non-specific IgG light chain that was detected by the secondary antibody (sheep anti-mouse IgG) in both WT and KO mouse kidneys. This IgG light chain band and unglycosylated PrP always overlapped, which affected quantitative analysis of the PrP band detected. Previous studies showed that most PrP in peripheral tissues is N-terminally truncated [14], corresponding to the C-terminal PrP fragment termed C1 [14,33]. We therefore treated the samples with PNGase F, a glycoaminidase capable of cleaving asparagine and N-acetylglucosamine residues in order to focus on quantitative analysis of the deglycosylated C1 without significant interference from the IgG band. We observed that a small amount of PrP migrating at approximately 18 kDa corresponding to C1 was barely detectable in the sham WT kidney, while the intensity of this band was significantly increased in the IR-injured kidneys after one or two days of reperfusion (*p* < 0.05 or *p* < 0.01; Fig 3B and 3C); the level of PrP^C^ in IR-injured kidneys after three days of reperfusion was similar to that of untreated kidney (*p* > 0.05) (Fig 3B and 3C). One-way ANOVA exhibited high differences in the means of PrP levels among the four groups of WT mice (sham and three groups of IR-injured mice, *p* < 0.001) or among the three groups of IR-injured mice (*p* < 0.005). Loading of individual samples on the gels was monitored using a protein concentration assay.

### Oxidative stress markers are increased in KO compared to WT kidneys

**Fig 4:**
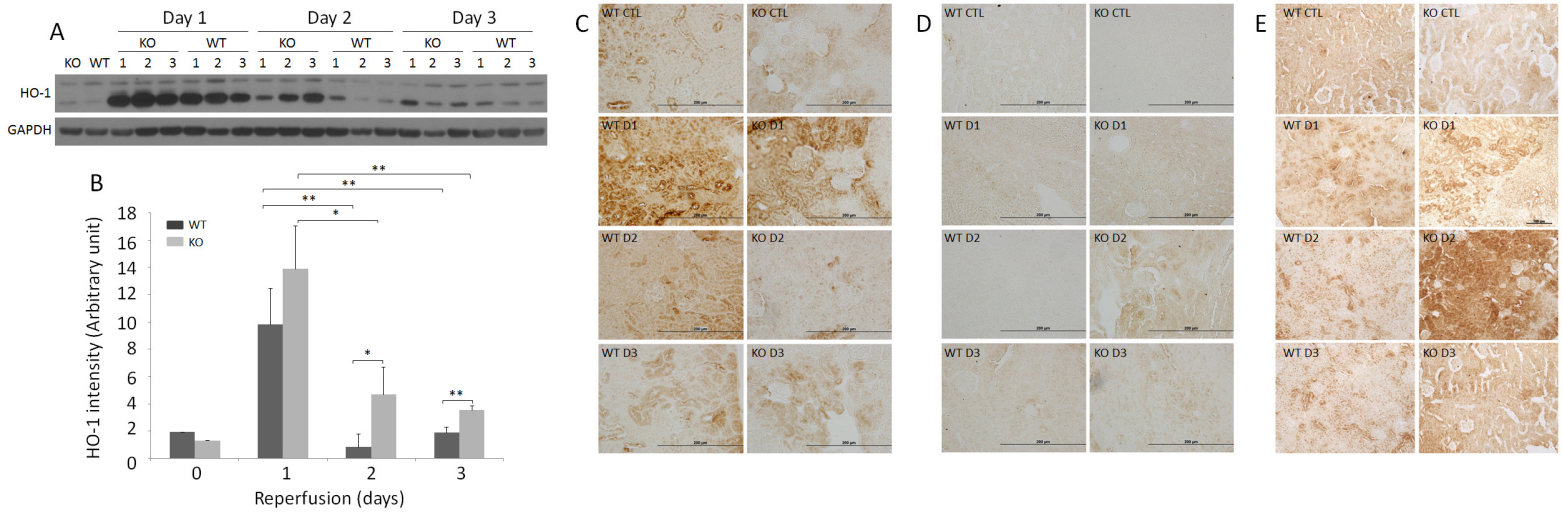
Detection of HO-1, nitrotyrosine and CML of sham and IR-injured WT and KO kidneys. (A) Detection of HO-1 from the frozen renal tissues of IR-injured WT and KO mice (three each) by Western blotting. The equal amounts of total proteins from renal homogenates were loaded based on BCA protein assay and the amounts of samples loaded were monitored by determining GAPDH. The Western blots are representative of three experiments. (B) Bar graph of HO-1 intensity based on quantitative analysis of the HO-1 band by densitometric analysis. The intensity of the protein band was normalized with GAPDH band intensity in each corresponding land. *p < 0.05; **p < 0.01. (C) HO-1 immunohistochemistry of sham and IR-injured WT and KO mice. IHC staining images are representative renal tissue sections from three mice of each group (bar = 200 μm; original magnification, x 400). (D) Nitrotyrosine immunohistochemistry of sham and IR-injured WT and KO mice. IHC staining images are representative renal tissue sections from three mice of each group (bar = 200 μm; original magnification, x 400). (E) CML immunohistochemistry of sham and IR-injured WT and KO mice. IHC staining images are representative renal tissue sections from three mice of each group (original magnification, x 200).

Heme oxygenase-1 (HO-1) has been observed to be upregulated by stress, and is believed to mediate transient resistance to oxidative damage from IR injury [34]. Using both Western blotting and immunohistochemistry, the levels of HO-1 were determined in sham and IR-injured kidneys. Western blot analysis revealed significant increases in the HO-1 levels on day 1 in the kidneys of injured WT and KO mice compared to sham WT and KO mice (*p* < 0.001). HO-1 in WT kidneys decreased to the amounts similar to sham mice by days 2 and 3 (Fig 4A and 4B). Although HO-1 in the KO kidney also diminished on days 2 and 3 compared to day 1, the KO mice still exhibited significantly higher HO-1 than WT (Fig 4A and 4B). The difference in the mean value of HO-1 was highly significant among all IR-injured kidneys of both WT and KO with different reperfusion times, as analyzed by the one-way ANOVA (*p* < 0.001). Consistent with the Western blot, immunohistochemistry revealed extremely intense HO-1 staining in kidney tubules in KO and WT mice on day 1; however, in contrast to the Western blot, more intense HO-1 staining was generally observed in WT than in KO kidneys of sham and all IR-injured mice (Fig 4C).

Nitrotyrosine is a marker of peroxynitrite generation [35–37]. Renal accumulation of nitrotyrosylated proteins has been reported in the setting of increased oxidative stress after IR injury [38,39]. Using immunohistochemistry, nitrotyrosylated proteins were analyzed on the kidneys of both WT and KO mice. Nitrotyrosine staining was mainly observed in tubular structures, and was more intense in KO than in WT kidneys, especially on day 2 (*p* < 0.05) (Fig 4D).

N^ε^-(carboxymethyl)lysine (CML) is a well-characterized glycoxidation product that accumulates in tissues after oxidative stress. CML is generated by the oxidation of both carbohydrates and lipids and acts as a biomarker for general oxidative stress [40,41]. We determined CML levels in the kidney following IR using immunohistochemistry. CML staining was mainly observed in renal tubular cells (Fig 4E). The intensity of the CML staining was greater in KO than in WT kidneys, particularly on day 2 (*p* < 0.05), whereas it was only slightly higher in KO than in WT on days 1 and 3 (Fig 4E).

### Mitochondrial respiratory chain complexes I and III are significantly decreased in KO compared to WT kidneys

**Fig 5:**
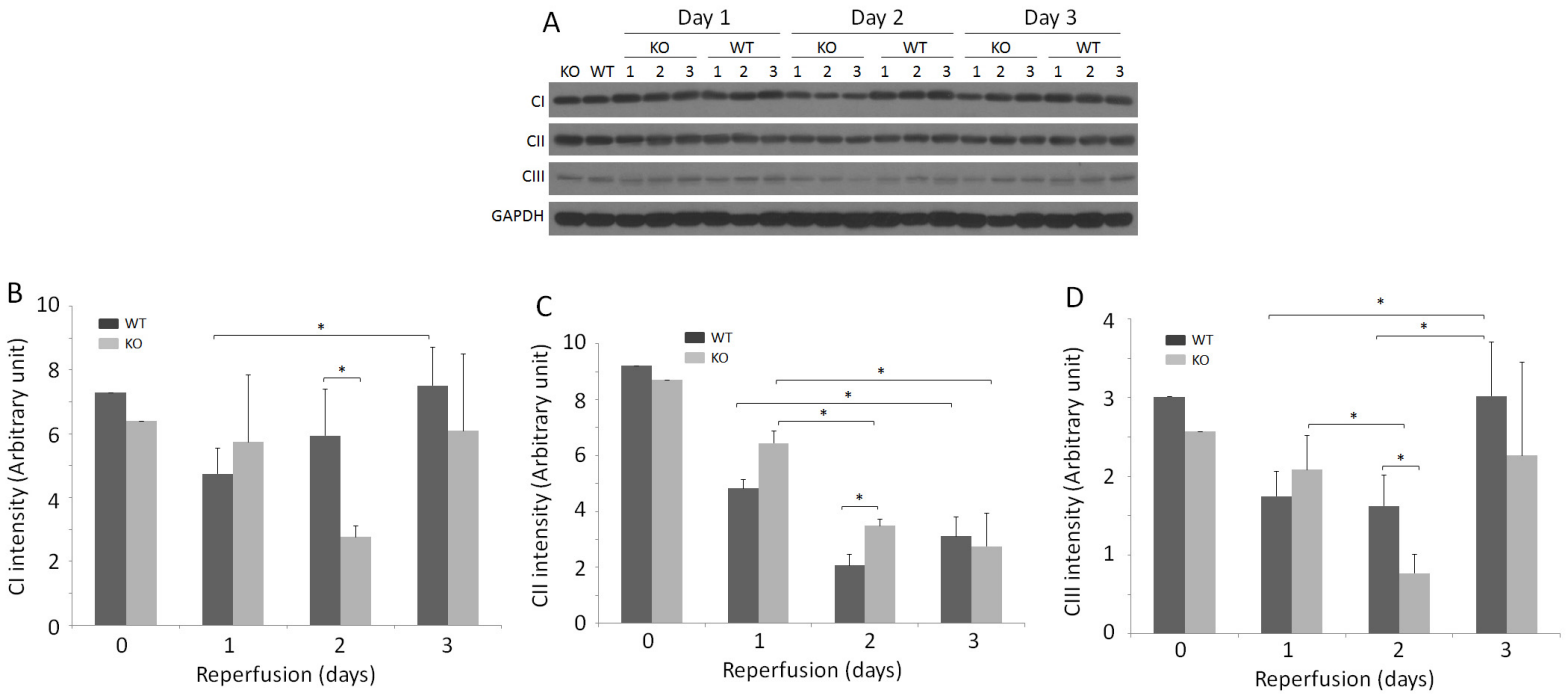
Detection of CI, CII and CIII of frozen kidney tissues of sham and IR-injured mice. (A) Detection of CI, CII, and CIII of renal homogenates from sham and IR-injured WT and KO mice by Western blotting. The equal amounts of total proteins from renal tissue homogenates were loaded based on BCA protein assay and the amounts of samples loaded were monitored by determining GAPDH. The Western blots are representative of three experiments. (B) Bar graph of mitochondrial respiratory chain complex CI intensity based on quantitative analysis of CI band from the kidney of sham and IR-injured mice by densitometric analysis. The intensity of the protein band was normalized with GAPDH band intensity in each corresponding land. *p < 0.05. (C) Bar graph of mitochondrial respiratory chain complex CII intensity based on quantitative analysis of CII band from the kidney of sham and IR-injured mice by densitometric analysis. The intensity of the protein band was normalized with GAPDH band intensity in each corresponding land. *p < 0.05. (D) Bar graph of mitochondrial respiratory chain complex CIII intensity based on quantitative analysis of CIII band from the kidney of sham and IR-injured mice by densitometric analysis. The intensity of the protein band was normalized with GAPDH band intensity in each corresponding land. *p < 0.05.

Renal tubular cells, especially proximal tubular cells, are extremely rich in mitochondria, which can readily undergo morphological changes following IR injury [42]. We investigated CI, CII, and CIII in sham and IR-injured WT and KO kidneys by Western blotting (Fig 5A). The levels of CI and CIII were lower on days 1 and 2 but not on day 3, in both WT and KO kidneys (Fig 5A through 5D). The levels of both CI and CIII were significantly lower in KO than in WT on day 2 (*p* < 0.05) (Fig 5A, 5B and 5D). While the levels of CII were dramatically decreased in all IR-injured kidneys compared to sham kidneys, they were higher in KO than in WT on day 2 (Fig 5A and 5C). The one-way ANOVA also indicated that the differences among all IR-injured groups in the means of CI or CIII were significant (*p* < 0.05) and highly significant in the means of CII (*p* < 0.005).

### Phosphorylated ERK1/2 is significantly increased in IR-injured KO and WT kidneys

**Fig 6:**
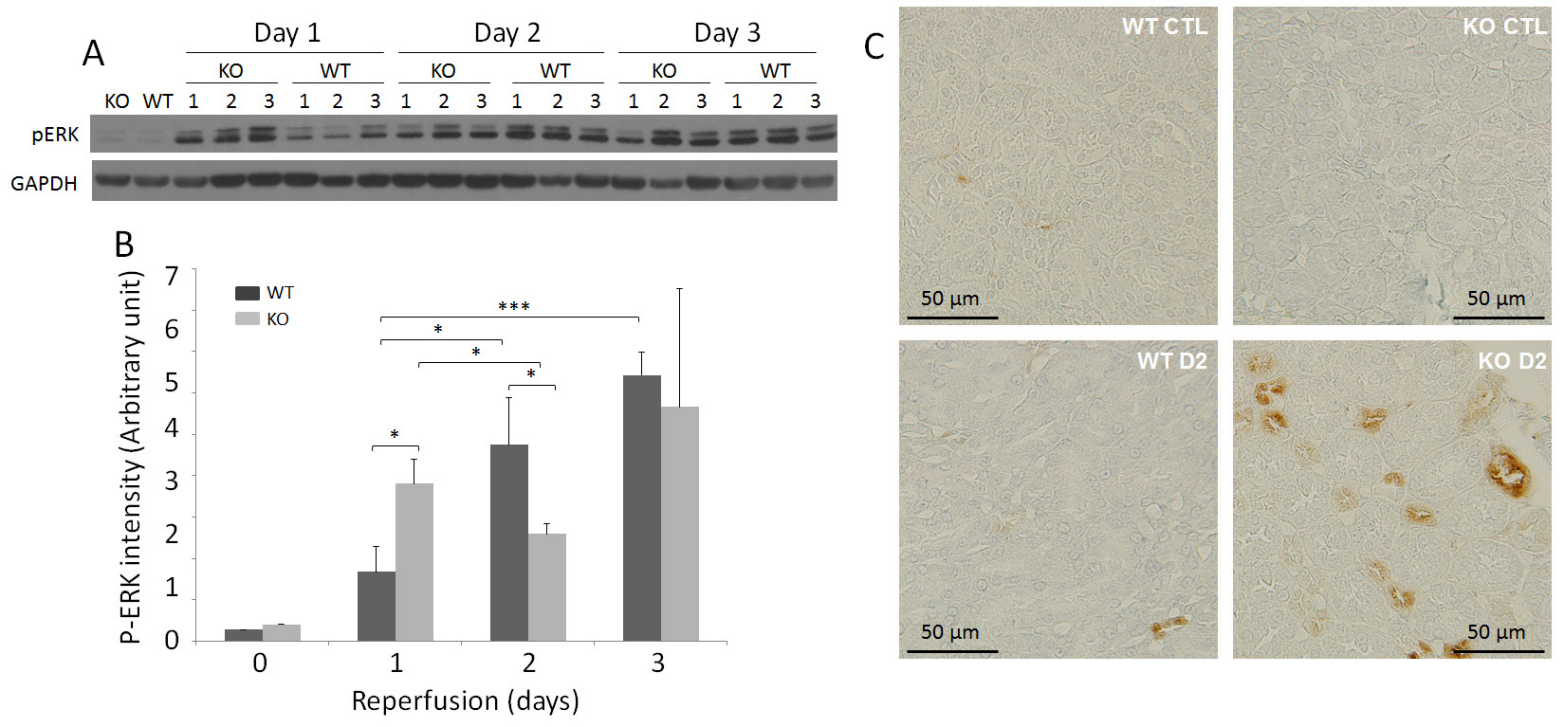
Detection of pERK of sham and IR-injured WT and KO kidneys. (A) Detection of pERK of renal homogenates from sham and IR-injured WT and KO mice by Western blotting. The equal amounts of total proteins from renal tissue homogenates were loaded based on BCA protein assay and the amounts of samples loaded were monitored by determining GAPDH. The Western blots are representative of three experiments. (B) Bar graph of pERK intensity based on quantitative analysis of pERK band from the kidney of sham and IR-injured mice by densitometric analysis. The intensity of the protein band was normalized with GAPDH band intensity in each corresponding land. *p < 0.05; ***p < 0.005. (C) pERK immunohistochemistry of sham and IR-injured WT and KO mice. Immunohistochemistry (IHC) staining images are representative renal tissue sections from three mice of each group (bar = 50 μm; original magnification, x 400).

To further explore the mechanisms underlying the potential protective role of PrP in renal IR injury, we investigated phosphorylated ERK1/2 (pERK1/2), which is often activated in the IR-injured brain [43–45]. Compared to the levels of pERK1/2 in the sham kidney, Western blotting revealed that the pERK1/2 levels in both WT and KO kidneys were significantly increased (Fig 6A and 6B). While the levels of pERK were observed to increase continuously from day 1 to day 3 in injured WT kidneys, notably, compared to day 1, the levels of pERK were decreased on day 2 but increased again on day 3 in injured KO kidneys (Fig 6A and 6B). KO exhibited a higher pERK level than WT on day 1, whereas on days 2 and 3, pERK levels diverged in opposite directions. The differences in the means of pERK were highly significant among the IR-injured WT kidneys (*p* < 0.005) but not significant among the three groups of IR-injured KO kidneys (*p* > 0.05), indicated by the one-way ANOVA. Remarkably, immunostaining of pERK was observed mainly in the renal tubular cells of KO mice subjected ischemia and reperfusion for 2 days (Fig 6C).

## Discussion

The following significant findings were made in our current study on WT and PrP KO mice subjected to IR-induced renal injury. First, the levels of serum creatinine were much higher in PrP KO than in WT mice. Second, the KO kidneys displayed more severe renal damage than WT. Third, the levels of PrP in the IR-injured WT kidney were increased compared to sham mice. Fourth, levels of HO-1 detected by Western blotting were higher, while HO-1 immunostaining in tissue sections was lower, in KO than in WT kidneys. Fifth, the immunostaining of nitrotyrosine and CML was more intense in KO than in WT kidneys. Sixth, mitochondrial respiratory chain complexes I and III were significantly lower in KO than in WT. Finally, immunostaining of phosphorylated ERK (pERK) was predominantly confined to tubular cells of KO mice, while the levels of pERK were significantly higher in WT than KO kidneys. All the above changes were most evident on day 2, one of the three examined reperfusion time points. These findings raise several issues and implications as to the role of PrP^C^ and the signaling pathways it may be involved in during renal IR injury.

The levels of serum creatinine have been widely used to monitor renal dysfunction during and after renal IR-injury [2,46,47]. We observed substantial increases in serum creatinine in both IR-injured WT and KO mice compared to sham mice, indicating that the IR protocol successfully generated AKI in our animals. While renal dysfunction was found in all mice subjected to IR-injury at the three time points, the significant differences in the levels of serum creatinine between WT and KO mice were only observed on day 2. Remarkably, the finding that day 2 is a critical time point was echoed by other changes including renal structural damage, oxidative stress, mitochondria dysfunction and activation of the ERK pathway found in our study, which all pointed to day 2 as the key time point at which the protective role of PrP^C^ in IR-induced renal injury is highlighted. At this time point, for instance, the highest PrP^C^ levels were detected in the IR-injured WT kidneys. Compared to WT kidneys, more severe tubular damage including patchy tubular injury and tubular cell apoptosis/necrosis was also observed in H&E stained KO kidney tissue sections on day 2. The time-dependent increase of PrP^C^ expression and its association with lesion severity have been found in the ischemic animal brains as well [27,25]. It is worth noting that the levels of serum creatinine were lower in KO than in WT mice even in the absence of IR injury, albeit no statistical significance was reached. The possibility that PrP^C^ plays a role in the renal function involved in creatinine clearance cannot be ruled out.

HO-1 is the rate-limiting enzyme that catalyzes heme degradation to biliverdin and ultimately to bilirubin, liberating carbon monoxide and free iron in the process [48,49]. It is upregulated in proximal tubular cells in response to oxidant stress [50,51] and confers dramatic cytoprotective and anti-inflammatory effects upon activation [50–52]. Our Western blotting and immunohistochemistry studies revealed a rapid increase in the levels of HO-1 in the WT and KO kidney on day 1. Notably, while Western blotting exhibited higher levels of HO-1 in KO than WT, immunostaining of HO-1 in renal tissue sections showed the opposite findings. Although we do not currently have a definite explanation for this discrepancy, it cannot be ruled out that Western blotting may detect an inactive HO-1 while immunohistochemistry detects its active form. If this was the case, it is conceivable to expect that PrP^C^ may be involved in activating HO-1. Since the WT mice exhibited less renal dysfunction and structural damage than KO mice, PrP^C^ deletion may fail to activate HO-1 to prevent oxidative stress injury of tubular structures. As a result, more oxidative stress markers including nitrotyrosine and CML were observed in KO than in WT kidneys on day 2, consistent with previous observations of more severe renal damage in KO than in WT at this time point. These findings strongly suggest that deletion of PrP^C^ results in a profound loss of anti-oxidative stress capability of the KO kidney. Interestingly, Morimoto et al reported that intense staining of CML and pentosidine, two well-characterized advanced glycation products formed under oxidative stress, was observed within renal tubular epithelial cells in HO-1 deficient patients [53]. Our immunohistochemistry indeed revealed less intense HO-1 staining but more intense CML and nitrotyrosine staining in the KO kidney compared to those in the WT kidney on day 2. Therefore, it is most likely that generation of active HO-1 may be hampered by deletion of PrP^C^, resulting in a decreased ability to protect renal tubules from IR-induced oxidative injuries.

Mitochondria are proposed to be the key generators and primary targets of oxidative stress [42]. The mitochondrial electron transport system is associated with the formation of superoxide anions leading to increased production of hydrogen peroxide by superoxide dismutase [54,55]. The enhanced production of superoxide anions and hydrogen peroxide was proposed to result from reduced electron flow through the mitochondrial respiratory chain, specifically through the inhibition of complex I (CI) or complex III (CIII) [56]. Moreover, reduced mitochondrial CIII is considered to be one of the major sites of superoxide production in the respiratory chain [57]. Our present study revealed that IR injury decreased the levels of CI, CII, and CIII in both WT and KO kidneys. Particularly, the levels of CI and CIII were significantly lower in KO than in WT, while the levels of CII were considerably higher in KO than in WT on day 2. This finding suggests that increased oxidative stress found in the KO kidney compared to WT on day 2 may be mainly associated with a decrease in CI and CIII.

Interestingly, ERK has been found to be a target of PrP^C^ signaling not only in neurons but also in non-neuronal cells [58]. Spudich et al observed that ERK was most rapidly activated in PrP-KO brains and further proposed that activation of the ERK1/2 pathway is causally involved in neuronal death in ischemic PrP-KO mice [28]. However, Shyu et al reported that the protective role of PrP^C^ overexpression in ischemic rat brains was blocked by intracerebral injection of an inhibitor of mitogen-activated protein kinase of ERK1/2 [27], suggesting that such a protective role of PrP^C^ in the ischemic brain is associated with activation of the ERK pathway. Given the proposal that the ERK signaling pathway has dual roles in IR-induced tissue injury [43], the discrepancy between the two studies may suggest that PrP^C^ plays a role in determining whether the ERK pathway has a beneficial or detrimental effect in IR injury. It has been proposed that the beneficial effects of ERK1/2 on the ischemic brain are associated with its involvement in growth factors, estrogen, preconditioning, and hypothermia, while the detrimental effects of ERK1/2 activity are associated with inflammation and oxidative stress [43]. This may be relevant to the kidney as well. It was reported that ERK mediates apoptosis of renal epithelial cells after exposure to oxidants or nephrotoxicants [59–63]. Consistent with this idea, the ERK pathway was further demonstrated to mediate mitochondrial dysfunction and necrosis induced by oxidant injury [63]. However, activation of the ERK pathway accelerates the repair of renal tubular epithelial cells and inhibits the progression of fibrosis following IR injury or unilateral ureteral obstruction [48,64]. It was striking that pERK staining was mainly confined to the tubular cells of IR-injured KO kidneys on day 2, suggesting that the ERK pathway is actively involved in renal tubular damage of KO mice. It is worth noting that the levels of pERK were radically decreased in KO kidneys compared to those of WT, as evidenced by Western blotting; however, more tubular staining of pERK was demonstrated by immunohistochemistry in KO kidneys on day 2. This unorthodox behavior of pERK and downstream of PrP^C^ deletion-associated activation of ERK pathway in the KO kidneys warrants further investigation.

## Materials and Methods

### Ethics Statement

All procedures involving animals were approved by the Case Western Reserve University IACUC and were in compliance with the Guide for the Care and Use of Laboratory Animals; these guide-lines were established by the Institute of Laboratory Animal Resources and approved by the Governing Board of the U.S. National Research Council.

### Animals

Prion protein knockout mice (KO) with the FVB background were originally obtained from the laboratory of Dr. George Carlson at the McLaughlin Research Institute (Great Falls, MT). FVB wild-type (WT) mice were obtained from Charles River (Spencerville, Ohio). They were maintained under specific pathogen-free conditions. Both KO and WT mice used in the experiments described in this study were ~5–10 weeks old and weighed ~20–30 g. All of the experiments were performed in accordance with the guidelines of Case Western Reserve University animal use regulations and approved by the institutional animal care and use committee at Case Western Reserve University. Sufficient actions were considered for reducing pain or discomfort of subjects during the experiments.

### Reagents

Primary antibodies used in this study included: mouse monoclonal antibody 6D11 against prion protein (Abcam, Cambridge, MA), rabbit monoclonal antibody against phosphorylated extracellular signal-regulated kinase ½ (pERK1/2) (Abcam, Cambridge, MA), rabbit monoclonal antibody against heme oxygenase-1 (HO-1), rabbit monoclonal antibody against GAPDH (Cell signaling), mouse anti-Total OXPHOS Human WB Antibody Cocktail (Abcam). Secondary antibodies including sheep anti-mouse IgG antibody and donkey anti-rabbit IgG antibody were purchased from GE Healthcare Life Sciences. TUNEL kits were ordered from Millipore (Billerica, Massachusetts).

### Renal ischemia/reperfusion (IR) injury model

Induction of kidney IR injury was performed as described previously [65]. In brief, mice were anesthetized with an intraperitoneal injection of ketamine (55 mg/kg) and xylazine (15 mg/kg). Following abdominal incisions, renal pedicles were bluntly dissected. The right ureter was ligated and cut proximately, and a microvascular clamp was placed on the right renal pedicle for 30 min. Mice were kept well-hydrated with warm saline and housed at a constant temperature of 32°C. After removal of the clamp and ligation of right renal pedicle, the right kidney was removed. The microvascular clamp was placed on the left renal pedicle for 30 min and mice were kept at 32°C. After removal of the clamp, the incisions were sutured, and the animals were allowed to recover with free access to food and water. In the sham operation group, the non-ischemic kidneys were subjected to the same protocol described above. All operations were performed in a blinded fashion. Mice were sacrificed on 0, 1, 2, or 3 days after reperfusion; blood samples were collected at the same time, and the left kidneys were harvested. Half of each kidney was frozen in dry ice and kept at -80°C and another half was fixed at 10% saline-buffed formalin for protein chemistry or histology analysis.

### Histological examination and immunostaining

Renal tissue samples harvested from mice on days 1, 2, or 3 post-reperfusion were fixed in 10% PBS-buffered formalin and then embedded in paraffin. Five-micrometer sections were stained with H&E by standard methods [66]. The tubular damage in KO and WT mice was assessed by a renal pathologist in a blinded fashion. Immunohistochemistry for expression of pERK1/2, HO-1, nitrotyrosine, and CML in renal tissue sections was performed as previously described [66].

### Assessment of renal function

Serum creatinine levels were measured using commercially available kits (BioAssay Systems, Hayward, CA) following the manufacturer's instructions or in the University Hospitals Case Medical Center Laboratory Service (Cleveland, Ohio) to assess renal function.

### Apoptosis assay

Apoptotic cells in IR-injured kidneys were detected by terminal deoxynucleotidyl transferase—mediated uridine triphosphate nick-end labeling (TUNEL) assays using the In Situ Cell Apoptosis Detection Kit I (Millipore, Billerica, Massachusetts) as described previously [67].

### Western blotting

Levels of PrP, pERK, HO-1, mitochondrial respiratory chain complexes I, II, and III in renal tissues were detected by Western blotting as reported previously [66]. The 10% kidney homogenates (w/v) were prepared in 9 volumes of 1X lysis buffer containing protease inhibitors (Roche, Indianapolis, IN) and phosphatase inhibitors (Pierce, Rockford, IL) (20mM Tris, 150 mM NaCl, 1% Nonidet P-40, 1% deoxycholate, 1% Triton X-100, 2mM EDTA, pH 7.4) by homogenization of kidney tissues with pestle on ice. Protein concentrations were determined with a protein assay kit from Bio-Rad (Hercules, CA) following the manufacturer's instructions.

### Statistic analysis

Results are expressed as means ± s.d. The statistical differences between two groups were analyzed by an unpaired Student’s t-test (two-tailed), while the one-way analysis of variance (ANOVA) was used to compare means for multiple groups or between group pairs. A value of *p* < 0.05 was considered statistically significant.
