# Correction: Prion Protein Protects against Renal Ischemia/Reperfusion Injury

**DOI:** 10.1371/journal.pone.0141025

**Published:** 2015-10-15

**Authors:** Bo Zhang, Daniel Cowden, Fan Zhang, Jue Yuan, Sandra Siedlak, Mai Abouelsaad, Liang Zeng, Xuefeng Zhou, John O'Toole, Alvin S. Das, Diane Kofskey, Miriam Warren, Zehua Bian, Yuqi Cui, Tao Tan, Adam Kresak, Robert E Wyza, Robert B. Petersen, Gong-Xian Wang, Qingzhong Kong, Xinglong Wang, John Sedor, Xiongwei Zhu, Hua Zhu, Wen-Quan Zou

There are errors in the author affiliations for Fan Zhang. The affiliations should appear as shown here:

Fan Zhang^2, 13, 15^

**2** Department of Pathology, Case Western Reserve University/University Hospitals Case Medical Center, Cleveland, Ohio, United States of America, **13** Department of Neurosurgery, Shandong University, Shandong provincial hospital, Jinan, The People’s Republic of China, **15** Department of Neurosurgery, Chengdu First People’s Hospital, Chengdu, The People’s Republic of China.
